# A Forma Correta de Identificar a Má Aptidão Cardiorrespiratória: Olhar Para a Direita

**DOI:** 10.36660/abc.20230630

**Published:** 2023-10-16

**Authors:** Willian R. Menegazzo, Anderson Donelli da Silveira

**Affiliations:** 1 Hospital de Clínicas de Porto Alegre Porto Alegre RS Brasil Hospital de Clínicas de Porto Alegre, Porto Alegre, RS – Brasil; 2 Universidade Federal do Rio Grande do Sul Programa de Pós-Graduação em Cardiologia e Ciências Cardiovasculares Porto Alegre RS Brasil Universidade Federal do Rio Grande do Sul - Programa de Pós-Graduação em Cardiologia e Ciências Cardiovasculares, Porto Alegre, RS – Brasil

**Keywords:** Disfunção Ventrículo Direito, Aptidão Cardiorespiratória, Infarto do Miocárdio, Reabilitação Cardíaca, Teste de Esforço/métodos, Ecocardiografia Doppler/métodos

O ventrículo direito (VD), anteriormente conhecido como ventrículo esquecido, vem ganhando cada vez mais atenção como forte indicador de pior prognóstico em doenças cardíacas.^1,2^ Além das implicações prognósticas, a disfunção do VD está associada à pior aptidão cardiorrespiratória (ACR), independentemente de outras variáveis anatômicas e funcionais.^3,4^ Infelizmente, as estratégias atuais de tratamento para a insuficiência do VD não conferem os mesmos benefícios clínicos e prognósticos da insuficiência do ventrículo esquerdo (VE),^5,6^ o que justifica novas estratégias de tratamento.

Ramirez et al.,^7^ acessaram esse importante tópico estudando a função do VD em 109 pacientes com infarto agudo do miocárdio com supradesnivelamento do segmento ST (IAMCSST), sua associação com ACR e sua melhora após reabilitação cardíaca (RC) em um centro em Monterrey, México. Seu objetivo principal foi avaliar a associação entre a função do VD e a ACR nesta população, e os objetivos secundários foram analisar a ACR antes e depois de um programa de RC em pacientes com ou sem disfunção do VD. A tolerância ao exercício foi avaliada através do teste de esforço cardiopulmonar, a função longitudinal do VD por meio da medida ecocardiográfica da onda S’ Doppler do anel tricúspide e da excursão sistólica do plano do anel tricúspide e a função radial do VD por meio da variação da área fracionada do VD. A população era predominantemente do sexo masculino (83%), com IAMCSST da parede anterior (55%), com mediana da fração de ejeção do VE de 49% e com função normal do VD (90%). Eles demonstraram associação e correlação entre disfunção radial e longitudinal do VD e ACR antes e depois da RC. O principal achado do estudo é que a disfunção do VD, seja radial ou longitudinal, após IAMCSST e antes da RC, foi associada à ausência na melhora de pelo menos 1 Equivalente Metabólico (MET) após um programa de RC (p = 0,028 para disfunção radial do VD e p = 0,008 para disfunção longitudinal do VD). Os autores concluíram que a disfunção do VD em pacientes com IAMCSST está associada e correlacionada com pior capacidade de exercício e classe funcional antes da RC e que a RC melhorou a ACR. Isto ajuda a identificar um subgrupo de pacientes com maior risco de serem sintomáticos, com pior prognóstico e possivelmente melhores candidatos à RC.

O estudo de Ramirez et al.,^7^ merece alguns comentários. Primeiro, a amostra é pequena, especialmente de pacientes com disfunção do VD, o que poderia limitar compreensão real da associação da disfunção do VD e da ACR após IAMCSST. Em segundo lugar, as conclusões foram sem uma análise multivariada: existem variáveis concorrentes, como a função do VE, que também estão associadas à ACR, e não poderíamos concluir com precisão se apenas a disfunção do VD poderia explicar a má ACR e a ausência de melhora após a RC independentemente de outros fatores. Terceiro, mais recentemente, a deformação longitudinal (strain) da parede livre do VD é considerada uma variável superior na avaliação da disfunção do VD devido à sua capacidade de detectar disfunção precoce do VD e com menor variabilidade inter e intraobservador.^2,8^ A avaliação da disfunção do VD com este método ecocardiográfico poderia influenciar as conclusões.

De fato, algumas limitações poderiam dificultar a interpretação e aplicação das conclusões destes autores. No entanto, os resultados estão alinhados com outros estudos que confirmam a importância da função do VD nas doenças cardíacas e o impacto combinado da disfunção do VD e da ACR.^2,9^

Pesquisas futuras nesta área deveriam incluir a avaliação combinada do teste de exercício cardiopulmonar e da ecocardiografia para melhor compreender a fisiopatologia da disfunção do VD na limitação do exercício e outros mecanismos concorrentes que poderiam explicar uma pior ACR^10^ para reconhecer e tratar esta população adequadamente.
