# Supplementary material for: A Forma Correta de Identificar a Má Aptidão Cardiorrespiratória: Olhar Para a Direita
Source: Arq Bras Cardiol. 2023 Oct 16;120(9):e20230630. [Article in Portuguese] doi: 10.36660/abc.20230630 (PMC10586814; doi:10.36660/abc.20230630)
Supplement: Translation

# Translation: The Right Way to Identify Bad Cardiorespiratory Fitness: Look to the Right

**Authors:** Willian R. Menegazzo, Anderson Donelli da Silveira

**Affiliations:** 1 Brazil Hospital de Clínicas de Porto Alegre, Porto Alegre, RS - Brazil; 2 Brazil Universidade Federal do Rio Grande do Sul - Programa de Pós-Graduação em Cardiologia e Ciências Cardiovasculares, Porto Alegre, RS – Brazil

**Keywords:** Ventricular Dysfunction, Right, Cardiorespiratory Fitness, Myocardial Infarction, Cardiac Rehabilitation, Exercise Test/methods, Echocardiography Doppler/methods

The right ventricle (RV), previously known as the forgotten ventricle, is progressively gaining attention as a strong indicator of poorer prognosis in heart disease.^1,2^ Besides prognostic implications, RV dysfunction is associated with worse cardiorespiratory fitness (CRF), independently of other anatomic and functional variables.^3,4^ Unfortunately, the current treatment strategies for RV failure do not confer the same clinical and prognostic benefits of the left ventricle (LV) failure,^5,6^ which limits its management and warrants new treatment strategies.

Ramirez et al.^7^ accessed this important topic by studying the RV function on 109 patients with ST-elevation myocardial (STEMI), its association with CRF, and its improvement after cardiac rehabilitation (CR) in a single center in Monterrey, Mexico. Their primary goal was to evaluate the association between RV function and CRF in this population, and the secondary objectives were to analyze the CRF before and after a program of CR in patients with or without RV dysfunction. They evaluated exercise tolerance through cardiopulmonary exercise testing and RV longitudinal function through echocardiographic measurement of the S’ Doppler wave of the tricuspid annulus and the tricuspid annular posterior systolic excursion and RV radial function through RV fractional area change. The population was mainly male gender (83%), with anterior STEMI (55%), median LV ejection fraction of 49%, and normal RV function (90%). They have shown an association and correlation between RV radial and longitudinal dysfunction and CRF before and after CR. The main finding is that RV dysfunction evaluated after STEMI and before CR, being radial or longitudinal, was associated with a lack of improvement of at least 1 Metabolic Equivalent Task after a program of CR (p = 0.028 for RV radial dysfunction and p = 0.008 for RV longitudinal dysfunction). The authors concluded that RV dysfunction in STEMI patients is associated and correlated with poorer exercise capacity and functional class before CR and that CR improved CRF. This helps to identify a subgroup of patients with a higher risk of being symptomatic, with a worse prognosis and possibly better candidates for CR.

The study of Ramirez et al.^7^ warrants a few comments. First, there were a small number of patients, especially with RV dysfunction, which could limit the actual understanding of RV dysfunction and CRF after STEMI. Second, the conclusions were done without a multivariate analysis: there are competing variables, such as LV function, that are also associated with CRF, and we could not conclude with precision if only RV dysfunction could explain the bad CRF and worse improvement after CR. Third, nowadays, RV-free wall longitudinal strain is emerging as a superior variable when evaluating RV dysfunction because of its ability to detect subtle RV dysfunction and less inter and intraobserver variability.^2,8^

Evaluating RV dysfunction with this echocardiographic method might influence the conclusions. Indeed, some caveats could limit the interpretation and application of these authors’ conclusions. However, the results align with other studies confirming the importance of accessing RV function on cardiac diseases and the powerful and additive prognostic impact of RV dysfunction and CRF.^2,9^

Future research in this field should include a combined evaluation of cardiopulmonary exercise testing and echocardiography to better understand the physiopathology of RV dysfunction in exercise limitation and other competing mechanisms that could explain worse CRF^10^ to recognize and treat this population adequately.
